# Tag attachment innovation on spurdog (*Squalus acanthias*) reveals year‐round coastal association of pregnant females in northeastern Atlantic waters

**DOI:** 10.1111/jfb.16000

**Published:** 2024-12-25

**Authors:** Claudia Junge, Keno Ferter, C. Antonia Klöcker, Otte Bjelland, Jon Albretsen, Robert J. Lennox, Finn Økland, Romaric Jac, Hector Andrade, Ole Thomas Albert

**Affiliations:** ^1^ Havforskningsinstituttet (Institute of Marine Research) Bergen Norway; ^2^ Ocean Tracking Network Dalhousie University Halifax Nova Scotia Canada; ^3^ Norsk institutt for naturforskning (Norwegian Institute for Nature Research) Trondheim Norway

**Keywords:** archival, diel vertical migration, movement, northeast Atlantic, Norway, PSAT, shark

## Abstract

The spurdog (*Squalus acanthias* Linnaeus, 1758) is a globally distributed squaliform shark that has historically been overfished but is now recovering in the northeast Atlantic. Data series on spurdog movement and habitat use have been somewhat limited to research surveys due to challenges associated with electronic tagging. Here, we offer a revised attachment method for externally attached pop‐up satellite archival tags that was successful in long‐term deployments on pregnant females. Twenty‐one spurdogs were tagged in two fjord systems in western Norway for an average of 243 days and provided new details about their behaviour, demonstrating affinity for coastal habitat based on the pop‐up locations and recovery positions of the tags (84% within 40 km from tagging location), and depth–temperature profiles. It is likely that parturition therefore occurs in these coastal waters, making them important to the life cycle of this species. The realized depth niche of tagged individuals suggested that surveys may miss sharks if they do not cover the full water column because the sharks used large parts of the water column and spent much time in shallower waters, albeit with seasonal variations (deeper and shallower in winter and summer, respectively). Adoption of this tagging method and combination with movement data from acoustic transmitters will help to better resolve the behaviour of this species as it transitions from a species at risk to a managed fishery. Such studies will provide a more comprehensive understanding of the species' habitat requirements that will empower better informed protections against a return to the red list of threatened species.

## 1 INTRODUCTION

Animal tracking can reveal interesting behavioural patterns and help understand species' movement, especially of highly mobile species such as sharks (Hussey et al., 2015). Habitat and space use of aquatic animals is characterized by both horizontal and vertical movements for purposes such as foraging, sheltering, reproducing, escaping, or growing. These biogeographic patterns are determined in part by the overall habitat, i.e., the sum of the specific resources that are needed by organisms (Thomas, 1979), and constrained by specific habitat qualities such as temperature, salinity, light, oxygen, substrate, and prey and predator presence (see e.g., Jac et al., 2022). Sharks have a key role as secondary and tertiary consumers that move matter and energy across great distances within the ocean (Hammerschlag et al., 2019). However, the movement dynamics of sharks are often elusive and challenging to investigate, which has led to the increased use of electronic tagging to provide this information.

The medium‐sized benthopelagic shark, spurdog (*Squalus acanthias* Linnaeus, 1758), also known as piked and spiny dogfish, has dynamic movement patterns in three‐dimensional space. This dogfish's fine‐scale horizontal movements are not well resolved, but it is known to have variable scales of movement, from small‐scale site fidelity to large‐scale movements across entire seas and ocean basins (Hjertenes, 1980; McFarlane & King, 2003; Pawson et al., 2009; Templeman, 1976; Thorburn et al., 2015; Vince, 1991), such that a fragmentation into residential and migratory contingents in spurdog populations has been suggested for decades (Campana et al., 2007; Carlson et al., 2014; Ketchen, 1986; McFarlane & King, 2003; Rulifson, 2010). Spurdog is globally distributed, and in Europe, the northern limit of spurdog extends to Norway and Iceland (McEachran & Branstetter, 1984). There is very limited knowledge about the behaviour and distribution of this shark in Norway (but see Jac et al., 2022, Andrade et al., 2024) and the complex coastal landscape consisting of extremely deep coastal fjords, the offshore Norwegian Trench, and the relatively shallow North Sea make the area available to spurdogs highly dynamic and conducive to the formation of local populations with unique movement ecologies.

Spurdog is predominantly found in temperate waters of the Atlantic and Pacific Oceans, inshore and offshore of the continental and insular shelf and upper slopes (Compagno, 1984). The species is commonly found at depths between 10 and 300 m but has been recorded to depths of 900 m (Andrade et al., 2024; Compagno, 1984; Jac et al., 2022; Sagarese, Frisk, Miller, et al., 2014). Beyond broad ranges of depth and temperature use, often inferred from bottom trawl surveys (Dunn et al., 2013; Sagarese, Frisk, Cerrato, et al., 2014; Dell'Apa et al., 2016; Jac et al., 2022;), little was known about the fine‐scale habitat use and environmental niche of spurdog until recently (see Klöcker et al. (2024) following the methodology from this study). It was listed as Endangered in Europe according to the IUCN Red List (Ellis et al., 2015) and Vulnerable globally (Finucci et al., 2020), in large part due to having late maturity and slow gestation (18–22 months, one of the longest of any living vertebrate), and to aggregating in groups that are vulnerable to overfishing and bycatch mortality in nontarget fisheries (Albert et al., 2019; Burgess, 2002; Dell'Apa et al., 2015; ICES, 2023; Jones & Ugland, 2001; Pawson et al., 2009; Stehlink, 2007). Historical overexploitation in the northeast (NE) Atlantic led to a collapse of the spurdog stock in the late 1990s, however, in large part due to strict and effective regulations preventing fishing, it seems to have recovered and its status continues to improve (Albert et al., 2019; ICES, 2023; Pawson et al., 2009). Consequently, it was recently moved from Endangered to Vulnerable on the Norwegian national Red List (Hesthagen et al., 2021) and the International Council for the Exploration of the Sea (ICES) released catch advice for the NE Atlantic for the first time since 2009 (ICES, 2022). New knowledge about the movement and habitat use of spurdog in Norway would provide important data for informing the management of this species as it transitions from endangered to being actively fished in a recovering population state. In addition to fundamental uncertainty about the scales of horizontal movements in Norwegian waters, uncertainty about parturition of spurdog persists (Sulikowski et al., 2013). Identifying parturition sites would help managers recognize key areas for the development of early life history in large marine predators, allowing for the introduction of spatiotemporal regulations to safeguard populations (Sulikowski & Hammerschlag, 2023).

Pop‐up satellite archival tags (PSATs) are frequently used for movement studies on sharks offering continuous movement data which are fisheries‐independent and do not require the physical recapture of the shark or the species to exhibit high site‐fidelity. While PSATs have previously been used on spurdog (Carlson et al., 2014; Sulikowski et al., 2010), tag attachment and early tag loss have posed significant challenges. This is related to the anatomy of the shark and its swimming behaviour, e.g., the relatively thin dorsal musculature does not allow for intramuscular tether attachment using titanium darts or Domeier anchors, therefore identifying an improved attachment method for the tags and establishing an effective tagging technique was the primary goal of this study. Following development of such a method, we were interested in whether pregnant females, which are particularly important from a population perspective, undertake offshore migrations and what their movement reveals about possible parturition sites. Finally, we explored how the realized environmental niche obtained from archival telemetry data compares with catch‐associated depths and temperatures from existing bottom trawl and longline surveys, and how far the latter can provide realistic behavioural snapshots. Identifying spurdog occupancy at depth is paramount to inform the planning of spurdog abundance surveys to constrain sampling depth ranges and make the use of boat time more effective. To these ends, we developed and refined an attachment method for PSATs through the dorsal musculature tagging 21 pregnant female spurdogs captured in western Norwegian fjords. We then investigated the year‐round distribution of the sharks, inspected depth–temperature profiles and characterized their environmental niche in comparison with equivalent niches derived from bottom trawl and longline surveys.

## 2 MATERIALS AND METHODS

### 2.1 Tagging method

**FIGURE 1:**
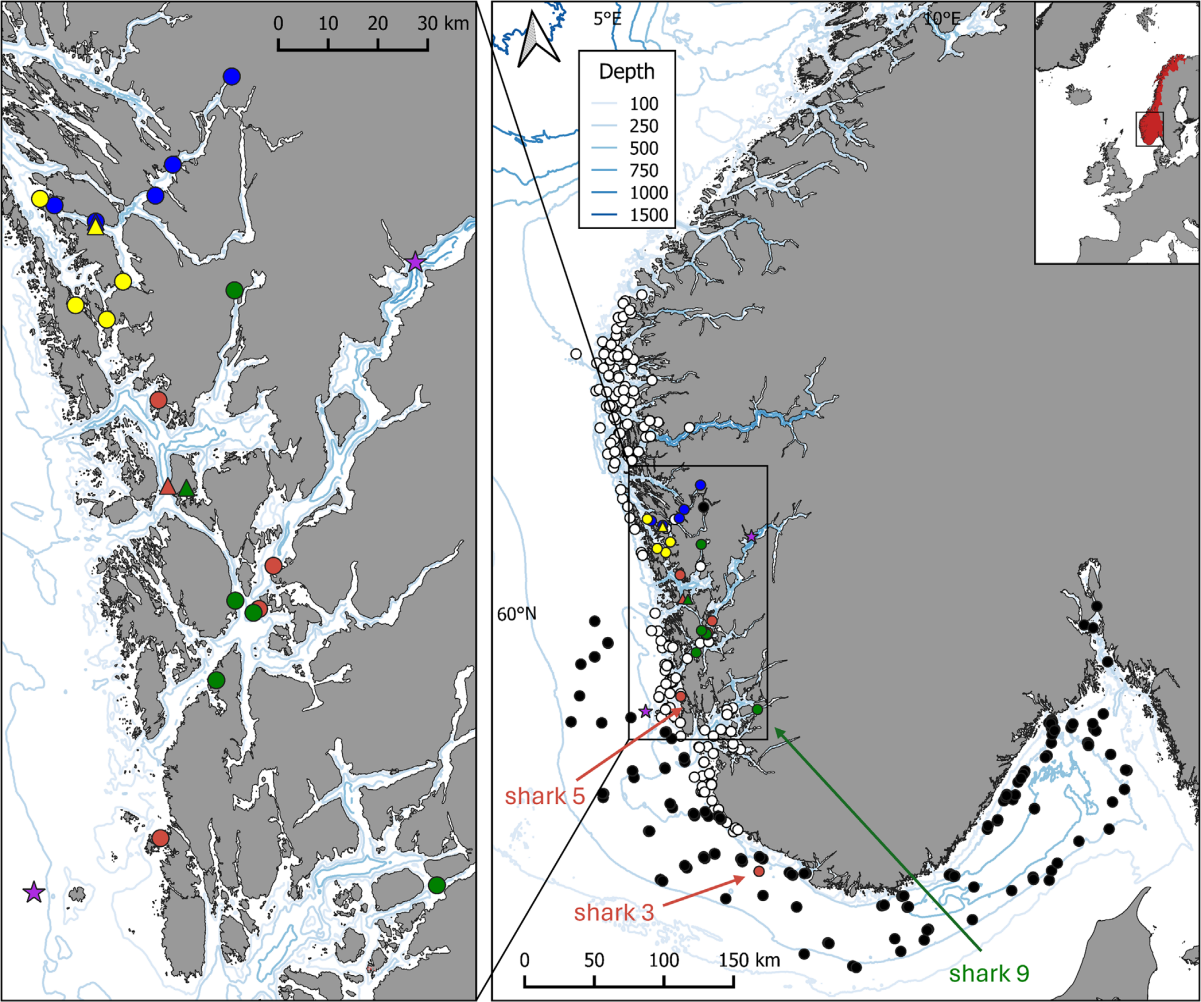
Map of the tagging (triangles) and pop‐off locations (circles) of female spurdogs (*Squalus acanthias* Linnaeus, 1758) (*n* = 19) along the southwestern Norwegian coast for the respective tagging years 2019 to 2022 (red, green, blue, yellow). Black circles indicate bottom trawl stations where spurdogs were caught during the bottom trawl survey (Reketokt) in January (2020–2023), and white circles stations where spurdogs were caught in the southern part during the spurdog bottom longline survey (Pigghåtokt) in autumn (2022–2023). The hydrographic stations Outer Utsira and H2 (inside the fjord system) are indicated by stars.

Spurdogs were tagged on the west coast of Norway (Figure 1) between 60.02°N and 60.52°N with pop‐up archival transmitting tags (PSATs, *n* = 21, depth sensor accuracy ±1% of reading, temperature sensor accuracy ±0.1°C, MiniPAT‐348; Wildlife Computers) in four consecutive years in November and December 2019 to 2022 (see Table 1). In 2019 and 2020, tagging took place south of the city of Bergen, in the Hardangerfjord area, whereas in 2021 and 2022 sharks were tagged north of Bergen, in Herdlefjorden (in the Osterfjord area, see Figure 1).

**TABLE 1:** Tagging information for all PSAT tagged female spurdogs (*Squalus acanthias* Linnaeus, 1758).

Shark	Tag ID	TL [cm]	DATEon	LOCon	DATEoff	LOCoff	REASON	Days	Days used
1	17P0023	95	27/11/2019	60.029, 5.338	25/05/2020	60.185, 5.333	Interval	180	179
2	17P0749	95	27/11/2019	60.029, 5.338	25/05/2020	59.791, 5.623	Interval	180	179
3	17P0796	87	26/11/2019	60.026, 5.336	24/05/2020	58.215, 5.959	Interval	180	180
4	17P0864	93	26/11/2019	60.028, 5.338	24/05/2020	59.866, 5.690	Interval	180	180
5	17P0866	97	26/11/2019	60.028, 5.338	24/05/2020	59.395, 5.196	Interval	180	116
6	20P2042	96	11/12/2020	60.022, 5.337	22/07/2021	59.670, 5.446	PD*	223	222
7	20P2044	87	08/12/2020	60.022, 5.337	26/06/2021	59.785, 5.602	PD*	200	199
8	20P2045	96	11/12/2020	60.022, 5.337	30/05/2021	60.370, 5.648	PD*	170	169
9	20P2046	92	12/12/2020	60.022, 5.337	20/04/2021	59.261, 6.152	Recapture	129	128
10	20P2047	91	12/12/2020	60.022, 5.337	15/07/2021	59.810, 5.541	PD*	215	214
11	20P2660	112	04/11/2021	60.518, 5.164	20/09/2022	60.554, 5.018	Recapture	320	319
12	21P0036	106	04/11/2021	60.518, 5.164	31/10/2022	60.608, 5.467	Interval	361	360
13	21P0037	117	04/11/2021	60.518, 5.164	29/01/2022	60.555, 5.392	Sensor error	86	82
14	21P0038	112	04/11/2021	60.518, 5.164	31/10/2022	60.757, 5.716	Interval	361	360
15	21P0039	109	04/11/2021	60.518, 5.164	31/10/2022	60.518, 5.164	Interval	361	360
16	21P2088	106	25/10/2022	60.518, 5.164	28/07/2023	60.37, 5.063	Const. pressure**	274	273
17	21P2202	107	25/10/2022	60.518, 5.164	25/10/2023	60.568, 4.967	Recapture	365	364
18	21P2203	115	25/10/2022	60.518, 5.164	26/10/2023	60.405, 5.244	Interval	366	364
19	21P2209	112	25/10/2022	60.518, 5.164	26/10/2023	60.339, 5.172	Interval	366	364
20	21P2204	116	25/10/2022	60.518, 5.164	NA	NA	Non report	NA	NA
21	21P2041	101	25/10/2022	60.518, 5.164	NA	NA	Non report	NA	NA

*Note*: Tag ID, dates of tagging and surfacing due to programmed pop‐off, early tag detachment or shark recapture (DATEon, DATEoff), total length (TL), coordinates of tagging and surfacing locations (LOCon, LOCoff), the reason for track termination and the number of days at liberty (DAYS) and those days used for analysis (DAYS used).

* Premature detachment.

** From 26.07.2023 where constant pressure triggered release and popoff 2 days later.

**FIGURE 2:**
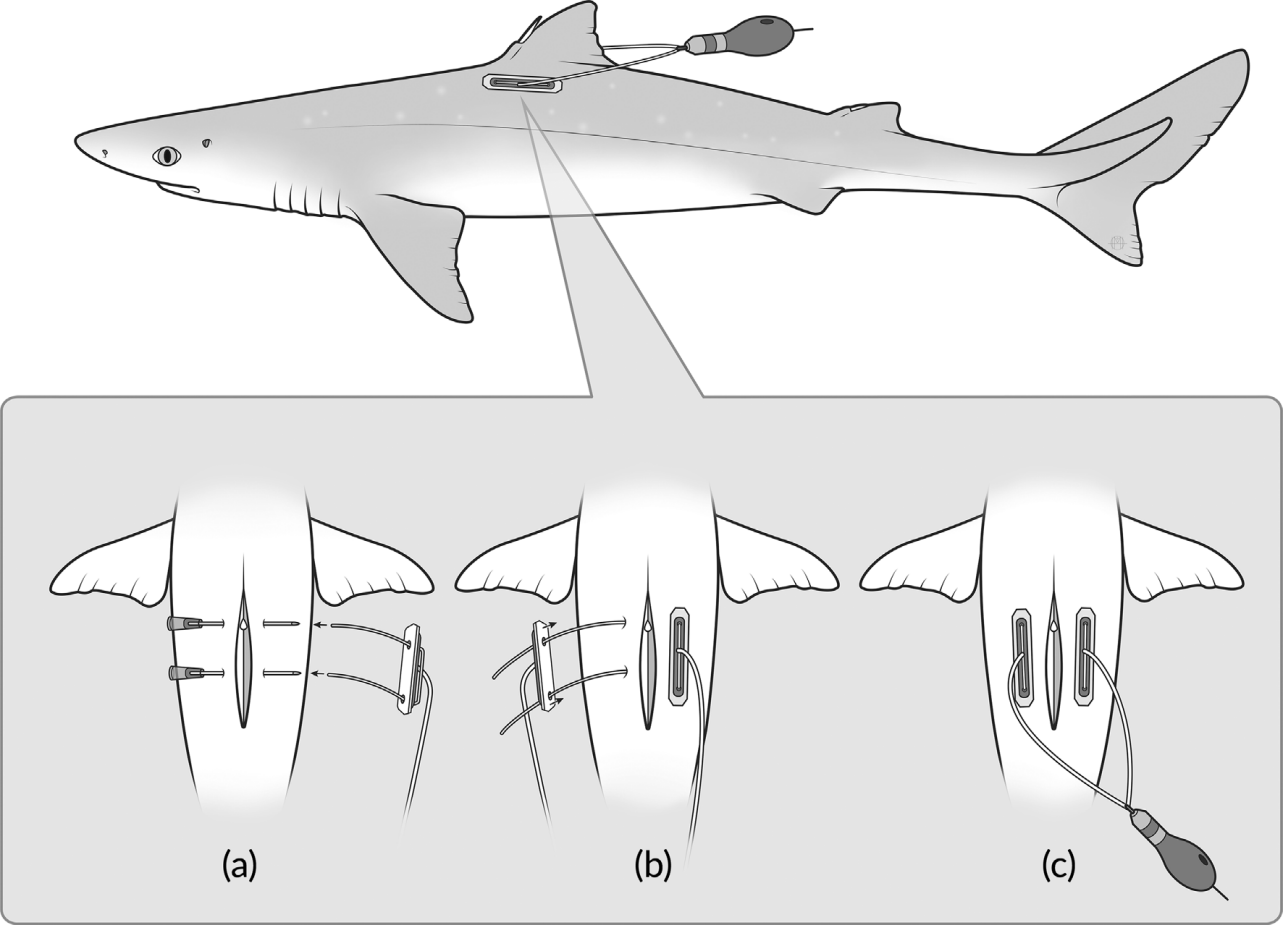
Illustration of the spurdog (*Squalus acanthias* Linnaeus, 1758) tagging method. The attachment setup and positioning are shown in the main picture and the attachment steps are shown in sequence in the detail images: (a) threading of the 0.8‐mm stainless‐steel wire through the musculature and positioning of the first plate below the first dorsal fin, (b) positioning of the second plate and therefore closing of the harness to which the PSATs were tied, and (c) full setup shown from the top.

Sharks were caught with rod‐and‐line using baited circle hooks and landed using a knotless landing net. Once on board, the individuals were dehooked and assessed for hooking injuries and bleeding. Only females in good condition, i.e., only lip‐hooked and no major bleeding, with a minimum total length (TL) of 85 cm were considered for tagging. These individuals were placed on a mattress covered with smooth nylon fabrics to avoid skin injuries, and a portable ultrasound (Mindray DP 50 vet, transducer model 75L50EAV) was used to determine if they were pregnant. Given the timing of the fieldwork, females were expected to either be towards the end of their second year of gestation, so either just before or just post‐partum, or at the end of their first pregnancy year. In 2019 and 2020, the aim was to tag females towards the end of their second year of their gestation based on foster size estimation from ultrasound images using the integrated measuring tool in the ultrasound. However, based on a later recapture with fosters (see results), this method was deemed as not reliable. Therefore, in 2021 and 2022, only females at the end of their first gestation year were tagged based on visible fosters during ultrasound examination (fosters are not visible just after fertilization) and the presence of yolk sacks (not present at the end of the second year of gestation). The anatomy and swimming behaviour of spurdog suggests that tag attachment should use a loop system, allowing the tag to move somewhat freely around the first dorsal fin. The loop should not be too large, posing an entanglement risk. Here, we tested the attachment method originally developed for eels from Økland et al. (2013). The PSATs were attached using two plastic plates (10 × 45 × 2 mm) fitted with 1‐mm thick braided nylon cord making up a harness to which the PSATs were tied (Figure 2). To prevent skin injuries, a 3‐mm thick silicone pad was placed between the plastic plates and the fish. The plastic plates were attached below the first dorsal fin (one on each side) and fixed using 0.8‐mm stainless‐steel wire which connected the plastic plates through the musculature of the fish (Økland et al., 2013; Figure 2a–c). The wires were inserted using two needles which were pushed through the musculature or cartilaginous base of the first spine from one side of the fish to the other. The wires were then inserted into the needles from the opposite side and pulled through the musculature together with the needles. The tags were allowed to trail freely behind the shark so that the harness and the tag were clear of the dorsal fin. In 2019 and 2020, the plates were placed slightly posterior to the first dorsal fin, whereas from 2021 onwards, the plates were positioned further forward (see results for explanation why), which constitutes the final tagging attachment positioning. Figure 2 shows the final attachment method.

### 2.2 Ethics statement

The capture of spurdogs was approved by the Norwegian Directorate of Fisheries. The care and use of the tagged animals complied with Norwegian animal welfare laws, guidelines, and policies as approved by the Norwegian Food Safety Authority (FOTS ID 20404 and 27484).

### 2.3 Tag retrieval and data processing

PSATs were programmed to pop off after 180 days in 2019, after 360 days in 2020 and 2021, and after 365 days in 2022. In the case of mortality (i.e., constant depth [variance: 2.5 m] over 2 consecutive days) or early tag detachment (i.e, floaters), the tags were programmed to (detach and) report after 2 days. In addition, tags were programmed to detach if the fish exceeded water depths of more than 1700 m. The home pinger was activated with an interval of 2 s in 2020–2022 to aid in tag recovery. Based on the estimated Argos locations of the tags, we used both visual search and search with a Goniometer (CLS RXG‐234; CLS Argos Systems) and a handheld radio receiver (ICOM IC‐R30) to physically recover the tags to obtain the full data archive of temperature, depth, light level, and triaxial accelerometer data at 5‐s resolution.

Data processing and analysis were performed in R (version 4.3.0). Archival PSAT data were visually inspected and cleaned to remove potential tagging and capture effects. While an inspection of the depth time series showed no indication of tagging effects on the diving behaviour, we conservatively removed the first 24 h of each track. We also removed any data indicating surface drifting or constant depth prior to tag release (Table 1). We could estimate the most probable tracks with the Global Position Estimator, version 3 software (GPE3; Wildlife Computers), but results were unreliable and are therefore not presented but discussed later. For further analysis, the raw data were aggregated to minute and hour intervals. Due to non‐normal distributions of depth and temperature data, both were aggregated using the median. Visualizations of the data were performed with *ggplot2* (Wickham et al., 2023).

### 2.4 Hydrographic profiles

To inspect how far the tagged individuals used environments inside or outside the fjord system, tag‐derived depth–temperature profiles were compared to conductivity, temperature, and depth (CTD) profiles from two hydrographic stations for two representative time periods in winter and autumn across the study period (IMR, 2023). Hydrographic stations comprised a station outside the fjord system, called Outer Utsira, and the H2‐station located in the center of the Hardangerfjord (Figure 1), which is deemed representative for the entire Hardangerfjord and adjacent fjords. Although the water exchange between offshore and inshore waters in the intermediate layers is relatively fast for such a large fjord system (e.g., Asplin et al., 2014), we know that there are time lags and seasonal biases in temperatures, here represented by measurements from Outer Utsira and Inner Hardangerfjord (H2). For comparison, available CTD profiles for winter (January and February) and autumn (October) between 2020 and 2023 were averaged for each available depth and standard deviations calculated. For the Utsira station these were 18 and 15 profiles for winter and autumn, respectively, while for H2 only four and five profiles were available. Tag‐based profiles were calculated based on 1‐min interval median depths and temperature values for corresponding time periods (winter = January and February, autumn = October). Depths were rounded to the next meter. For each meter with more than 60 data points the mean temperature and respective standard deviation was calculated across a given month or time interval. A rolling mean with a 5‐m window was applied to smooth the profiles.

### 2.5 Environmental niche analyses and comparison to survey data

The environmental niche used by the tagged sharks within the deployment period was compared with the environmental niche occupied by spurdogs caught in scientific surveys to establish an understanding of the temperature conditions prevailing within the study area and somewhat beyond (from 57 N to 62 N). For both the tagging and survey data, environmental niches were calculated as two‐dimensional kernel densities for depth and temperature pairs for months during the study period where survey data are available for the study area. Kernel densities were constructed using the MASS package (Ripley et al., 2023). We also calculated the 50% and 95% density contours to highlight core and overall niche spaces. The tag‐based niches were calculated based on hourly median depth and temperature records. In the case of survey data, station depths at which spurdog catches occurred were linked to temperatures extracted for the station location and date from the Norwegian Coastal Model, Norkyst. Norkyst applies the Regional Ocean Modelling System (ROMS; http://myroms.org, and see e.g., Shchepetkin & McWilliams, 2005) using 800 × 800 m resolution in the horizontal and 35 vertical levels, and is explained thoroughly in Asplin et al. (2020). Norkyst resolves both the Norwegian coastal waters and the offshore, northflowing Norwegian Atlantic Current and is very well suited for detailed hydrodynamic studies off the Norwegian coast.

Surveys included (i) the annual shrimp bottom trawl survey in January in the North Sea and the Skagerrak (2020–2023) and (ii) the annual spurdog longline survey in October along the Norwegian coast (2022–2023; Figure 1), both following a random‐stratified design. The longline survey comprises a mixture of randomly predefined stations to facilitate unbiased abundance estimates and supplementary stations including informant putative hotspots. All spurdogs caught are measured, weighed, sexed, and given maturity scores. Pup numbers are recorded for late‐pregnant females and the second dorsal fin spine is removed for age determination, as well as a fin clip for genetics (Andrade et al., 2024). Spurdogs were caught at depths between 17 and 312 m. The shrimp survey covers the Norwegian Trench and therein predefined stations with depths between 115 and 530 m (see e.g., Søvik et al., 2024; Søvik & Thangstad, 2021). All spurdogs which were caught were worked up in the same way as described above for the longline survey.

## 3 RESULTS

### 3.1 Tag retrieval and data processing

Nineteen tags were physically recovered after early (*n* = 5) and scheduled (*n* = 11) detachment, or recapture by fishers (*n* = 3) and their full archive was downloaded. Two tags from the 2022 tagging season never reported (Table 1). Due to premature tag loss after extending the programming to 360 days in 2020, the tag attachment plates were moved forward in 2021 so that one of the stainless‐steel wires was placed further forward on the shark's body, going through the cartilage just where the first dorsal spine starts. This decision was based on observations from recaptures and recovered tags from the 2020 tagging season, which still had the plastic plates and the harness attached to them. In several cases, the wire moved through the musculature, finally resulting in premature tag loss, which is why the attachment point was adjusted in the 2021 tagging season and used in 2021 and 2022.

All tags, apart from three, were found within a connected fjord system and within a maximum distance of 40 km from their original tagging location (Figure 1 and Table 1). Tags 17P0796 (shark 3) and 17P0866 (shark 5), both from the 2019 cohort, were found 205 and 71 km from their tagging locations, respectively, and tag 20P2046 (shark 9) from 2020, tagged in the same fjord system, was found 96 km from its tagging location. In 2019, all tags stayed on the sharks as programmed for 180 days. In 2020, when the scheduled pop‐off was extended to 360 days, the tags recorded data for between 129 and 223 days. For four of the tags, we suspect premature tag shedding leading to the detachment and floating of the tags to the surface from where we collected them. In 2021 and 2022, after having modified the tagging method, no such early tag loss was observed. In 2020 and 2021, a shark was caught after 129 and 86 days, respectively, and the tags were returned to us. Due to a sensor failure in tag 17P0866 (shark 5, 2019 cohort) and 21P0037 (shark 13, 2021 cohort), the track was terminated 5 days prior to the appearance of any extreme and implausible depth records, resulting in only 116 and 82 days, respectively, for subsequent analysis. From the 2022 cohort, one shark 21P2202 (shark 17) was captured 1 day prior to the programmed pop‐off date and one tag (21P2088, shark 16) recorded constant pressure and released after 275 days. Overall, analyzed data ranged from 82 to 364 days, with an average of 243 days (see Table 1).

### 3.2 Horizontal movement and hydrographic profiles

Due to the high latitude, highly dynamic fjord systems connected with offshore waters and with high mountain ridges influencing the light regime, robust geolocation estimates could not be obtained. The pop‐up locations, due to recaptures or early detachment, were distributed throughout the year. Based on those pop‐up locations, sharks 3, 5, and 9 left the Hardangerfjord system during their time at liberty.

**FIGURE 3:**
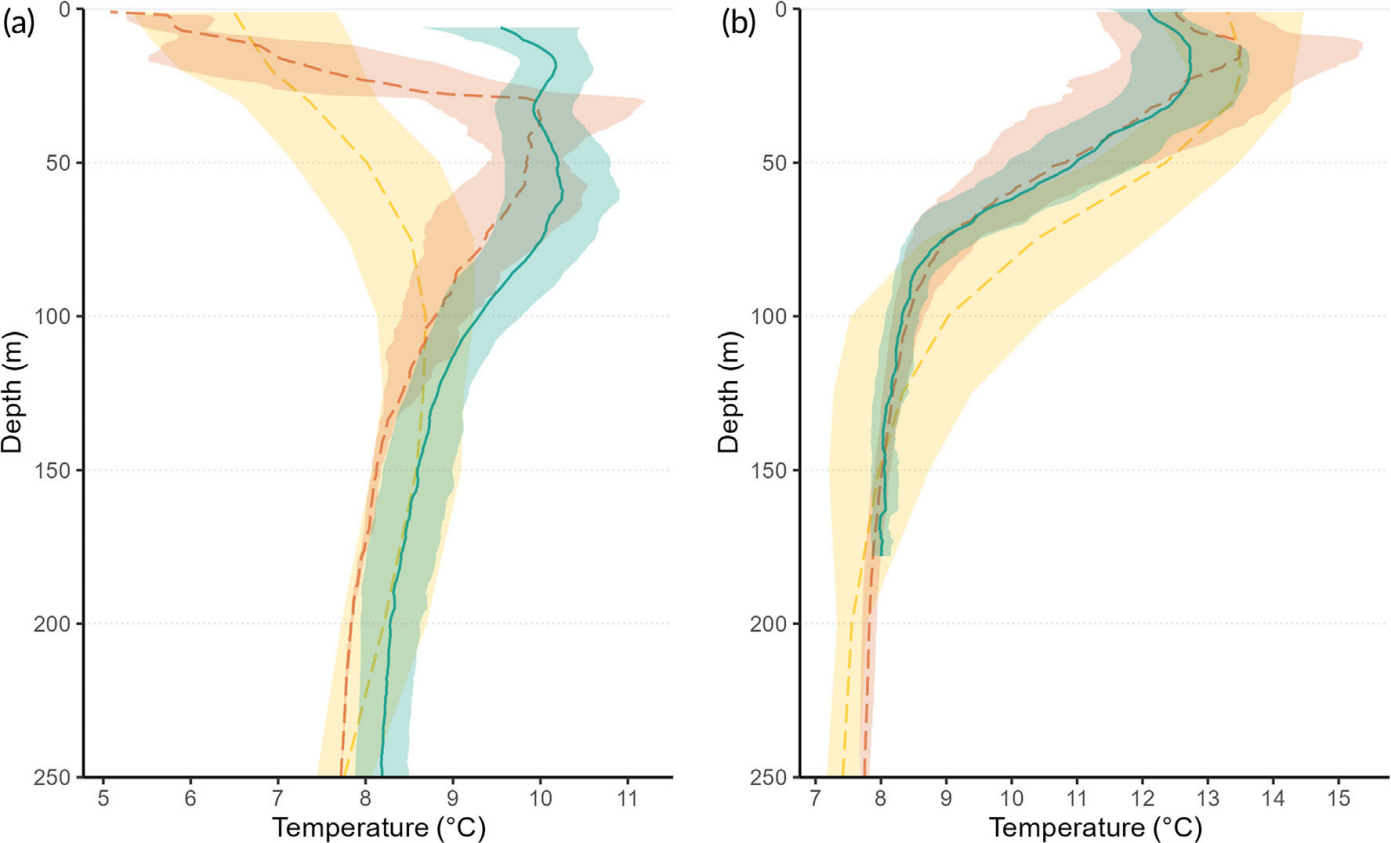
Depth–temperature profiles for the hydrographic stations and from the tracking data of spurdogs (*Squalus acanthias* Linnaeus, 1758). Hydrographic stations Utsira (yellow) and H2 (orange) as well as tracking data from used archival PSATs (turquoise) for (a) January and February (*n*_CTD_Utsira_ = 18, *n*_CTD_H2_ = 4, *n*_PATs_ = 19) and (b) October (*n*_CTD_Utsira_ = 15, *n*_CTD_H2_ = 5, *n*_PATs_ = 7) 2020–2023. Means and standard deviations of temperatures are shown for each meter (for the Utsira station, data were only available at 11 depths). Archival tracking data based on minutely median temperatures for each depth rounded to the next meter. The *y* axis is restricted to 250 m, corresponding to the depth for which CTD profiles were available.

Encountered depths and temperatures were similar to the oceanographic conditions available in the fjords systems, with depth–temperature profiles collated by the tags mimicking the CTD profiles at the H2 station, particularly in winter, where the conditions were distinct from the outside station Utsira (Figure 3). Sharks 3, 9, and 10 apparently encountered slightly different conditions in January and February (Figure S1).

### 3.3 Environmental niche

The 19 sharks occupied water with median temperatures of 9.4°C (7.7°C, 13.9°C), including 4.5 and 18.1°C as respective minimum and maximum median temperatures. The values in parentheses are temperatures at the 2.5% and 97.5% quantiles. Occupied temperatures varied both seasonally as well as daily. Median monthly values increased from 8.3°C (7.4, 10.5) in May to 14.0°C (10.7, 14.8) in September and consistently decreased again over the winter months (Figure S3).

The median depth used by the individuals was 63.3 m (5.0, 288.5), ranging between 0 and 577.5 m as the respective minimum and maximum depth. Median depths were greatest in January with 97.5 m (46.5, 299.0) and shallowest in August with 7.5 m (4.0, 19.0), as shown in Figure S3.

### 3.4 Environmental niche analyses and comparison to survey data

**FIGURE 4:**
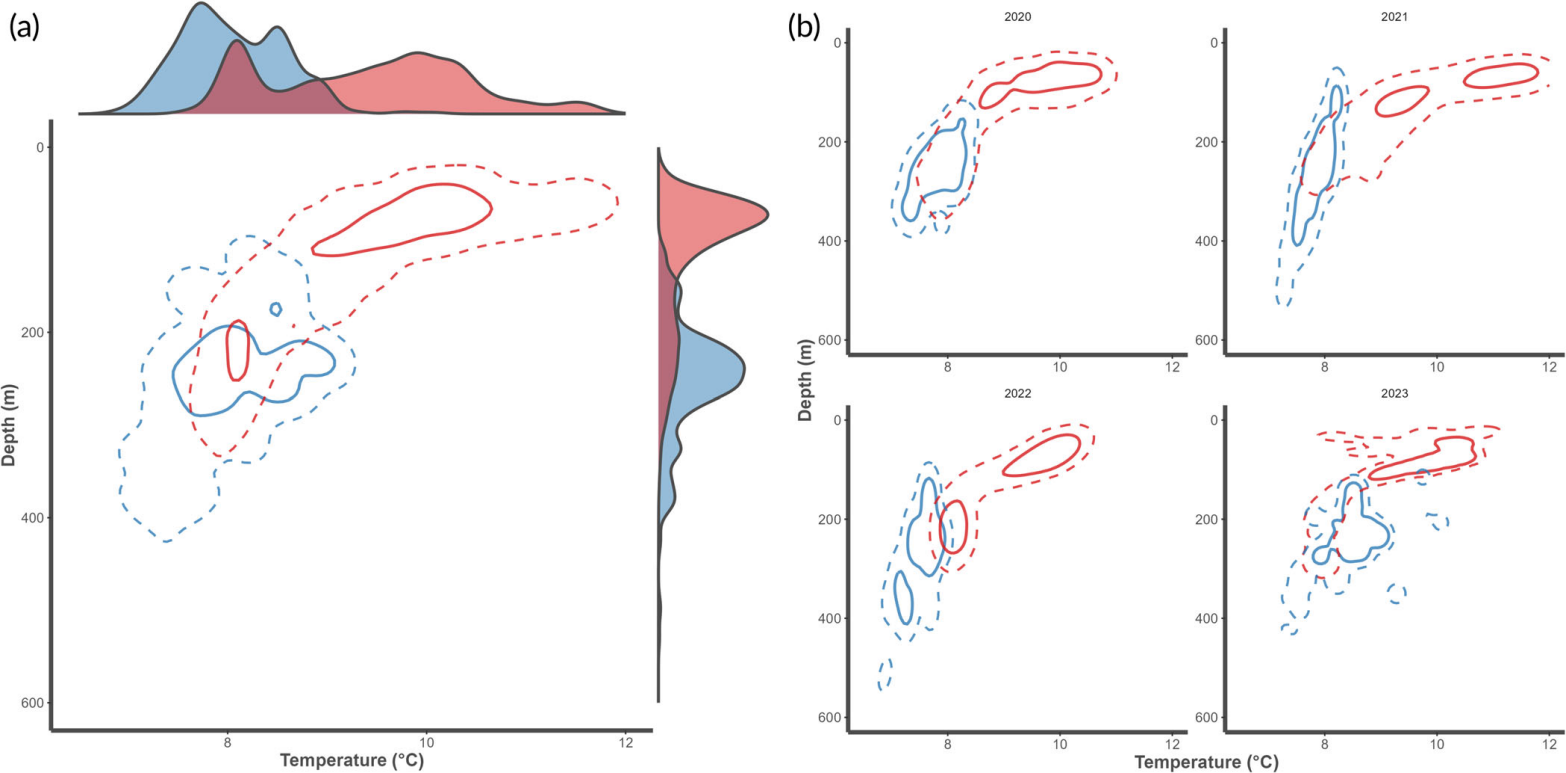
Spurdog (*Squalus acanthias* Linnaeus, 1758) depth–temperature niche based on kernel densities for median hourly records from obtained archival PSAT data (red, *n* = 13,991, this study) and extracted modelled temperatures for shrimp bottom trawl survey stations with spurdog catches (Reketokt, blue, *n* = 166) in January 2020–2023 (a) and split by the respective year (b). Solid and dashed lines indicate the niche space that encompasses 50% and 95% of the points, respectively. In (a), marginal densities are shown for both covariates, temperature (on top) and depth (right side).

Overall, tagging data suggested sharks occupied shallower and warmer waters compared to survey‐based data (bottom trawl and longline). This trend was particularly pronounced in the comparison with the catch data from the bottom‐trawl survey in winter, and less pronounced in the comparison with the catch data from the bottom longline survey in autumn. In January, high‐use areas in the depth–temperature space were between 30 and 300 m considering both the space use by the tagged individuals and the occupied space by the survey‐caught individuals (Figure 4a). The pattern for the tagged sharks was bimodal, with shallow depths of 30–110 m and temperatures between 9 and 11°C and deeper ones of 200–230 m at about 8°C. Sharks were predominantly caught at survey stations between 200 and 300 m in 7–9°C water, showing an overlap with the realized niche of the tagged sharks at around 200–230 m and 8°C (Figure 4). The overall pattern of the tagged individuals using shallower waters than those caught in the survey was consistent across years. Interannual differences in the overlap of the realized niche likely resulted from individual variation in habitat use as well as changes in the distribution of survey tows to encompass colder and deeper stations (Figure 4b).

**FIGURE 5:**
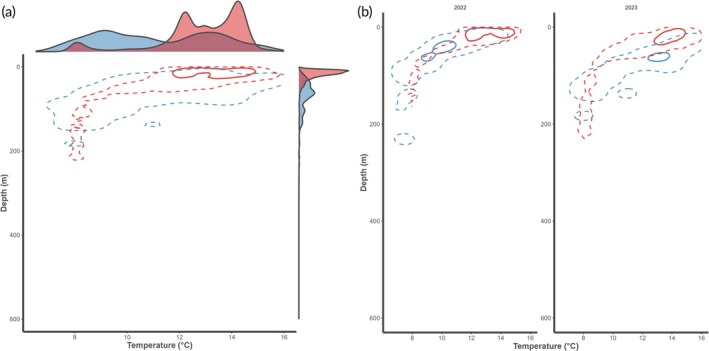
Spurdog (*Squalus acanthias* Linnaeus, 1758) depth‐temperature niche based on kernel densities for median hourly records from obtained archival PSAT data (red, *n* = 9194, this study) and extracted modelled temperatures for longline survey stations with spurdog catches (Pigghåtokt, blue, *n* = 1596) in October 2022–2023 (a) and split by year (b). Solid and dashed lines indicate the niche space that encompasses 50% and 95% of the points, respectively. In (a), marginal densities are shown for both covariates, temperature (on top) and depth (right side).

In autumn, the realized niche of the tagged sharks was unimodal, favoring shallow warm waters (<50 m, 12–15°C) coinciding with the surface layer of the fjord above the thermocline (and pycnocline) (Figure 5a). The sharks were caught rather equally distributed across 7–16°C and 10–200 m, with a slight preference around 80 m and 11°C. Interestingly, the catch depth was similar for the survey‐caught sharks in both years, 2022 and 2023, however the “core” thermal niche in which 50% of the catches occurred varied by 3°C, at about 10°C in 2022 and 13°C in 2023 (Figure 5b).

## 4 DISCUSSION

This study provides the first long‐term archival data for spurdog at such high resolution. PSATs offer fisheries independent data and do not require the physical recapture of the shark. While they have been used previously for spurdog (Carlson et al., 2014; Sulikowski et al., 2010), a longer‐term attachment of the tags has previously presented challenges due to the thin dorsal musculature not allowing for traditional tether attachment using titanium darts or Domeier anchors. The refined attachment methods developed as part of this study allowed us to obtain shark tagging data of more than 360 days without any early tag loss in the third and fourth years of the study. Using the detailed records of temperature and depth obtained from 19 individuals, movement extent and environmental niche use were examined.

### 4.1 Coastal association of pregnant females

Although spurdog can migrate long distances in the ocean (Templeman, 1976), pregnant females tagged in the Hardanger‐ and Osterfjord areas did not appear to make extensive movements to specific pupping or breeding grounds in the critical autumn/winter months (October to January), when pupping is expected. Given the temporal coverage of the tagging data with regards to the reproductive cycle, that is, a deployment covering the second half of the pregnancy, sometimes only partially, it remains unclear how far individuals might undertake smaller‐scale migrations, e.g., to the Norwegian trench for breeding purposes, which are much more difficult to detect in this ecosystem with this type of tag. Proximate offshore migrations of spurdog have been suggested by Carlson et al. (2014) for the northwest (NW) Atlantic. Given the high latitudes, steep mountains, and the highly variable and dynamic oceanographic conditions of the study region and the limited horizontal movements inferred from the pop‐off locations, PSATs turned out to be suboptimal tools to resolve the horizontal movements of these individuals. Although the likelihood scores of applied GPE3 models were high, the results did not fit well with the fact that most tags/recaptures occurred within the fjords or close to the coast. Double‐tagging of the last cohort with both PSATs and acoustic tags proved this as the fish were registered by acoustic receivers inside the fjord while the GPE3 model predicted that the fish were far offshore.

However, two lines of evidence suggest that the spurdogs did not move long distances: (i) the tags were all recovered within the same coastal area, within 40 km (84% of the sharks) and 205 km (16%) of the deployment site, and (ii) depth–temperature profiles of the tagged sharks resembled most closely CTD profiles at the inner fjord hydrographic station H2. Sharks 3 and 9 must have left the fjord system, based on the pop‐up locations. Those sharks show differences in their depth–temperature profiles in January and February compared to the H2 station, which indicates that this movement into adjacent fjord systems occurred in winter. A similar movement to more offshore locations might also have occurred for shark 10, which subsequently moved back into the same fjord system, given its tag pop‐up location. As conditions in autumn were more similar between inner and outer fjord stations, autumn profiles are therefore more difficult to assign to inner‐ or outer‐fjord conditions. Based on the findings in this study, there is no indication that pregnant females exhibit a seasonal offshore or north–south migration pattern, as suggested in earlier mark‐recapture studies in the NW and NE Atlantic (Aasen, 1964; Campana et al., 2007; Gauld & MacDonald, 1982; Hjertenes, 1980; Holden, 1965; Pawson, 1995; Rulifson, 2010; Vince, 1991). This might be linked to behavioural differences among sexes (Vince, 1991) or subpopulations (e.g., Campana et al., 2007; Ketchen, 1986; McFarlane & King, 2003; Templeman, 1976; Thorburn et al., 2015), or as a result of maturity or pregnancy status (e.g., Carlson et al., 2014).

Spurdog has a prolonged embryonic development period of about 18–24 months (e.g., Natanson et al. 2017) with parturition most likely between October and January in Norwegian waters (neonates in winter surveys, pers. comm). Parturition ostensibly coincides with the onset of the reproductive season, meaning that the winter months are important to the reproductive ecology of the species. New eggs develop during pregnancy, such that females are ready to breed again in the same winter after having given birth (Ford, 1921; Henderson et al., 2002; Jones & Geen, 1977; Jones & Ugland, 2001; Ketchen, 1972; Nammack et al., 1985; Stenberg, 2005). However, a study in the NW Atlantic found that mating occurs there in fall, either after giving birth or at the point of 1‐year pregnancy from the previous year's mating (Jensen 1965). Given that only females with embryos visible via ultrasound in November/December were tagged, we assume that the tracking period is likely to have covered the second year of the female's gestation period because it takes about 11 months until embryos reach about 10 cm in Nordic waters (Jones & Ugland, 2001). Thus, for individuals for which the tag remained attached until late October or early November (sharks 12, 14, 15, and 17–19), the pop‐up location might be indicative for parturition sites, providing evidence for coastal or even fjord‐based parturition sites on the west coast of Norway. Indeed, an area with a disproportionately high abundance of immature spurdogs, albeit at least already young‐of‐the‐year, was identified near the Espevær and Røvær islands (Andrade et al., 2024), in proximity to the southernmost pop‐up locations, but more data are needed to confirm whether such area might serve as a spurdog nursery area (Andrade et al., 2024; Heupel et al., 2007). This aligns with findings from the NW Atlantic, where neonate spurdogs were found in coastal association by trawling in New England, corroborating earlier findings suggesting regional movements for adult females along the Eastern Seaboard (Sulikowski et al., 2010, 2013). Earlier evidence from the British Isles found indication of aggregations of females in the eastern Celtic Sea to release their young, while also suggesting that pregnant as opposed to non‐pregnant mature females are found inshore (Pawson, 1995). More detailed studies on the movement dynamics of late‐pregnant and breeding females are needed to disentangle the complex spatio‐temporal dynamics and identify critical locations for the life history of spurdogs, such as parturition and breeding sites. Passive acoustic telemetry, allowing for high‐resolution horizontal movement patterns across long timescales, would likely be an effective method of identifying parturition areas for spurdogs in this system with higher confidence.

### 4.2 Environmental niche and comparison to survey data

The temperatures experienced by tracked sharks, ranging from 4.5 to 18.1°C (median = 9.4°C), are warmer and have a wider range than water temperatures reported for spurdogs caught during trawl surveys in Norwegian waters, 5.6–9.6°C (Jac et al., 2022), 7–13°C (this study) or the Bay of Fundy and Scotian Shelf (Shepherd et al., 2002). This comes as no surprise given that such trawl surveys deploy their gear at the bottom while spurdogs exhibit diel vertical migration behaviour (Klöcker et al., 2024) throughout a range of depths. Trawl surveys typically do not sample that far inshore, which results in deeper depths at most survey stations than those frequented by the tagged sharks. This is especially the case for the survey we used for comparison here, as the Reketokt is carried out in the Norwegian Deep, where it is deeper and also colder than the surrounding coastal waters. This comparison was, however, valuable as catch data from this survey are used as part of the spurdog stock assessment in ICES through its integration into a joint, international survey index (ICES, 2023). Bottom trawl surveys, however, are recognized to not sample spurdogs effectively due to the species' benthopelagic lifestyle and thus partial unavailability to trawls (Carlson et al., 2014; ICES, 2023) as well as such surveys being designed for other target species. Therefore, the spurdog longline survey was initiated in 2021 in Norway (Andrade et al., 2024). When comparing the realized environmental niche based on the tagged sharks with the one derived from the longline survey in autumn, it becomes clear that there is a high density of sharks in shallower waters beyond the longline survey's station design. Our results indicate that current stock indices heavily relying on data collected at deeper depths might fail to account for an important component of the population, underestimating spurdog biomass. Spurdog survey catchability is affected by environmental variables (seasonal, diel) and spurdog maturation stage (Sagarese et al., 2016), and these factors were not accounted for in our comparisons between tracked and survey data. However, despite differences in sampling methods (tracking, bottom trawl, longlines), the apparent deeper median depth used by spurdogs in the winter (January) compared to the autumn (August) is ubiquitous among methods and seems to confirm anecdotal fisher information reporting greater spurdog catches at depth during colder winter months, but in shallower waters during warmer autumn months. With regards to maturity, the preference for warmer, shallower water is also known for larger and female individuals in the NW Atlantic (Dell'Apa et al., 2014, 2016; Sagarese, Frisk, Cerrato, et al., 2014; Shepherd et al., 2002). Selection of warmer waters by adult females has been suggested as a strategy to elude adult males to avoid competition and harassment, a tactic that results in spatial segregation (Dell'Apa et al., 2014; Ford, 1921; Wearmouth et al., 2012; Wearmouth & Sims, 2008). However, it was recently shown that during the relative warm autumn in the southern Norwegian coast, no spurdog segregation occurred with regards to maturity, sex, or depth as most maturity stages were registered in the presence of the opposite sex, at both shallow and deep stations (Andrade et al., 2024). Prioritization of shallower stations (<150 m) is then advised for planning future autumn surveys.

Compared to existing tagging studies at northern latitudes using data storage tags or PSATs, recorded temperatures match well with reported ranges of 6.3–15.2°C (most common 10–11°C) along the Scottish west coast and 2.8–19.2°C (mean = 9.2°C) in the northern Gulf of Maine (Carlson et al., 2014; Thorburn et al., 2015). Depths ranging from 0 to 577 m (median = 63.3 m) also align well with corresponding studies (0–481.5 m, mean = 92.6 m) where deeper areas are available (Carlson et al., 2014) and also fall in the range described in earlier studies (Campana et al., 2007; Dell'Apa et al., 2015; Dunn et al., 2013). Considering available depths and the variable diving behaviour and depth use while at depth, tagged individuals spent a considerable amount of time off the bottom, representative of a pelagic habitat use, corroborating findings from the NW Atlantic (Carlson et al., 2014; Sulikowski et al., 2010).

### 4.3 Implications for informing fisheries management

Our study occurred during the last years that commercial fishing for spurdog was banned in Norway and extended into the first year (2023) with a re‐opened coastal fishery (with some restrictions). Additionally, spurdogs are commonly bycaught by commercial and recreational fishers, and their overall vulnerability to fishing is an important question, especially given the recent change in status in Norway and the recent re‐opening of a catch advice by ICES (ICES, 2022, 2024). Three of the tags (14%) were reported recaptured during the study. This is a promising recapture rate for future tagging programs, and that during a time where the spurdog fishery was closed in Norwegian waters and the rest of the NE Atlantic (except for the last 10 months in which one shark was recaptured), and hence all spurdogs were caught as bycatch. Now, after the re‐opening of the coastal fishery targeting spurdogs, it can be expected that more individuals will be caught and landed, which in return would lead to higher or at least similar recapture rates of tagged individuals, making the initialization of a large conventional tagging program worthwhile, not the least to follow the encounter rates through catch or bycatch. Conventional tagging programs such as those conducted in Canada in the mid‐1900s would provide valuable data on fishing vulnerability (Templeman 1984) and could cover a much larger sample size over a much longer time period. One important aspect to consider in such a program would be acquiring data for both males and females, given that we only tagged females and previous studies have indicated sex‐biased capture of males (Haugen et al., 2017). However, as there are no comparable size restrictions for conventional tagging due to much smaller tag sizes, tagging both females and males, as well as immature individuals, will not be an issue.

We found no indication of a seasonal offshore migration pattern, however, three sharks seemed to have moved out of the fjord system they were tagged in at some point during their time at liberty given the pop‐up locations of their tags. Given that spurdogs are currently managed as one unit within the NE Atlantic (ICES, 2022, 2023, 2024), further studies specifically addressing the scales of their movement on one side and potential smaller‐scale habitat association with fjord systems on the other side are needed to evaluate levels of population connectivity. High‐resolution horizontal data on various scales would help to reduce remaining uncertainties with regards to horizontal habitat use and elucidate underlying environmental cues, such as in the case of the observed high variability of depth use in spring. Observations from the British Isles show that tagged female spurdogs leave narrow seawater lochs in May after having resided there in winter months (Thorburn et al., 2015) and earlier fisheries data suggest mature females rapidly disperse from a winter aggregation site in late spring (Pawson, 1995). With literature suggesting limited transferability of presented results to males or different age classes and non‐pregnant females, we continue to lack a holistic view of the biotic mechanisms underlying habitat selection and niche segregation (Dell'Apa et al., 2015). Passive acoustic telemetry studies might help to elucidate underlying mechanisms in this dynamic system. Those usually exhibit a higher horizontal and temporal resolution and allow the tagging of smaller individuals, which could aid in disentangling sex and age class differences in space use. Ultimately, when combining tagging‐methods, including PSAT, acoustic and conventional, datasets could then be integrated into population dynamic models together with survey and fisheries‐dependent data to better inform stock assessment, as suggested by Senina et al. (2020).

### 4.4 Conclusions and outlook

Pop‐up satellite archival tags are a suitable tool with which to gather high‐resolution depth and temperature data from free‐swimming fish such as spurdog, especially when tags are recovered. The development of a new attachment method represents an important advancement that can support research throughout the global distribution of this shark as well as for other related squaliform dogfishes. At these latitudes and for individuals that do not conduct extensive migration, PSATs will not provide reliable horizontal movement data unless informed by, for example, acoustic detections for inputting known locations. The female spurdogs in this study exhibited a coastal association during time of parturition in the fjords of western Norway, a key finding for the population ecology and management of these sharks. Their preferences for shallow waters as shown here and their use of the entire water column and different depth zones shown in Klöcker et al. (2024) suggest that surveys may be challenged to effectively characterize the abundance of this species if the survey passes while the individuals are surface‐oriented. Therefore, the inclusion of pelagic stations in the relevant surveys might be a worthwhile consideration. As the spurdog stock rebuilds in the NE Atlantic and the fishery begins to accelerate, exploitation of this stock will increase and more data on the movement and ecology of spurdogs will be needed. Our study points to a need for a greater understanding of the species' association with the fjords, including long‐term residency and collective behaviour for such a gregarious animal. More knowledge about size, age, and sex segregation in movement dynamics will be valuable to fisheries managers to help identify important areas for reproduction and growth for vulnerable life stages and through aiding survey design which will deliver age and sex disaggregated data for stock assessments, together with population genetics, individual variation in movement behaviour, and availability to fishing gear. These questions emphasize the need for more fine‐scale tracking data in the NE Atlantic. As spurdog transitions from a species at risk back to an exploited species, advancements in tagging and attachment methods such as demonstrated in this study can support the next phase of management.

## AUTHOR CONTRIBUTIONS

C.J., K.F., and O.T.A. developed the concept. K.F. and O.B. tagged the sharks and refined the tagging method. F.Ø. provided the initial tagging method and attachments. C.J. led the project and acquired funding. C.J., C.A.K., K.F., and R.L. wrote the manuscript. C.A.K., J.A., R.J., and O.T.A. analyzed the data. C.J., K.F., C.A.K., R.L., J.A., O.T.A., and H.A. interpreted the results. All authors contributed to revisions of the manuscript, and all authors read and approved the final manuscript.

## FUNDING INFORMATION

Tagging in Norwegian waters was financed by the Institute of Marine Research (IMR) through the Norwegian Sea programme. IMR financed C.J., K.F., O.B., J.A., R.J., H.A., and O.T.A. C.A.K. was financed from the Norwegian Research Council as part of her PhD within the Sharks on the Move project (NRC #326879).

## Supporting information


**FIGURE S1.** Photograph of the tag attachment on the shark (shown for shark 12). ©Keno Ferter, Havforskningsinstituttet.
**FIGURE S2.** Depth–temperature profiles for the hydrographic stations and from the tracking data of spurdogs (*Squalus acanthias* Linnaeus, 1758). Hydrographic stations Utsira (yellow) and H2 (orange) as well as tracking data from archival PSATs for sharks 3, 9 and 10 for 2020–2023. Means and standard deviations of temperatures are shown for each meter (for Utsira station data were only available at 11 depths). Archival tracking data based on minutely median temperatures for each depth rounded to the next meter. The *y* axis is restricted to 250 m, corresponding to the depth for which CTD profiles were available.
**FIGURE S3.** Depth–temperature profiles for the hydrographic stations and from the tracking data of 19 spurdogs (*Squalus acanthias*). Hydrographic stations Utsira (yellow) and H2 (orange) as well as tracking data from archival PSATs (turquoise) for each month (from 1 = January to 12 = December) within the deployment period (November 2019–October 2023). Means and standard deviations of temperatures are shown for each meter (for Utsira station data were only available at 11 depths). Archival tracking data based on hourly median temperatures for each depth rounded to the next meter. The *y* axis is restricted to 250 m, corresponding to the depth for which CTD profiles were available.

## Data Availability

The tracking data that support the findings of this study are available from the corresponding author (C.J.) upon reasonable request until December 2025; after that, they will be made public with the following DOI: 10.21335/NMDC‐832456473.
