# Supplementary material for: Tag attachment innovation on spurdog (Squalus acanthias) reveals year‐round coastal association of pregnant females in northeastern Atlantic waters
Source: J Fish Biol. 2024 Dec 25;106(5):1654–67. doi: 10.1111/jfb.16000 (PMC12120337; doi:10.1111/jfb.16000)
Supplement: Supplementary file 1 — FIGURE S1. Photograph of the tag attachment on the shark (shown for shark 12). ©Keno Ferter, Havforskningsinstituttet. FIGURE S2. Depth–temperature profiles for the hydrographic stations and from the tracking data of spurdogs (Squalus acanthias Linnaeus, 1758). Hydrographic stations Utsira (yellow) and H2 (orange) as well as tracking data from archival PSATs for sharks 3, 9 and 10 for 2020–2023. Means and standard deviations of temperatures are shown for each meter (for Utsira station data were only available at 11 depths). Archival tracking data based on minutely median temperatures for each depth rounded to the next meter. The y axis is restricted to 250 m, corresponding to the depth for which CTD profiles were available. FIGURE S3. Depth–temperature profiles for the hydrographic stations and from the tracking data of 19 spurdogs (Squalus acanthias). Hydrographic stations Utsira (yellow) and H2 (orange) as well as tracking data from archival PSATs (turquoise) for each month (from 1 = January to 12 = December) within the deployment period (November 2019–October 2023). Means and standard deviations of temperatures are shown for each meter (for Utsira station data were only available at 11 depths). Archival tracking data based on hourly median temperatures for each depth rounded to the next meter. The y axis is restricted to 250 m, corresponding to the depth for which CTD profiles were available. [file JFB-106-1654-s001.docx]

**Supporting Information**


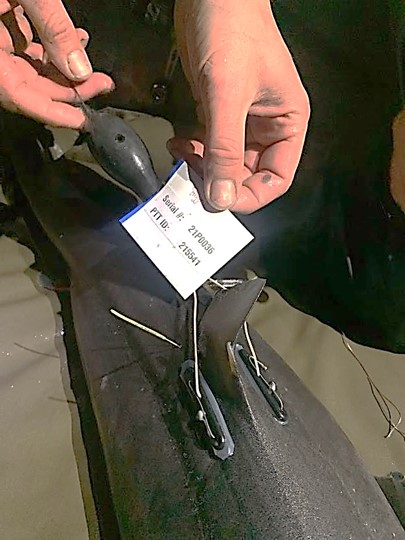


Figure S1: Photo of the tag attachment on the shark (shown for shark 12). ©Keno Ferter, Havforskningsinstituttet


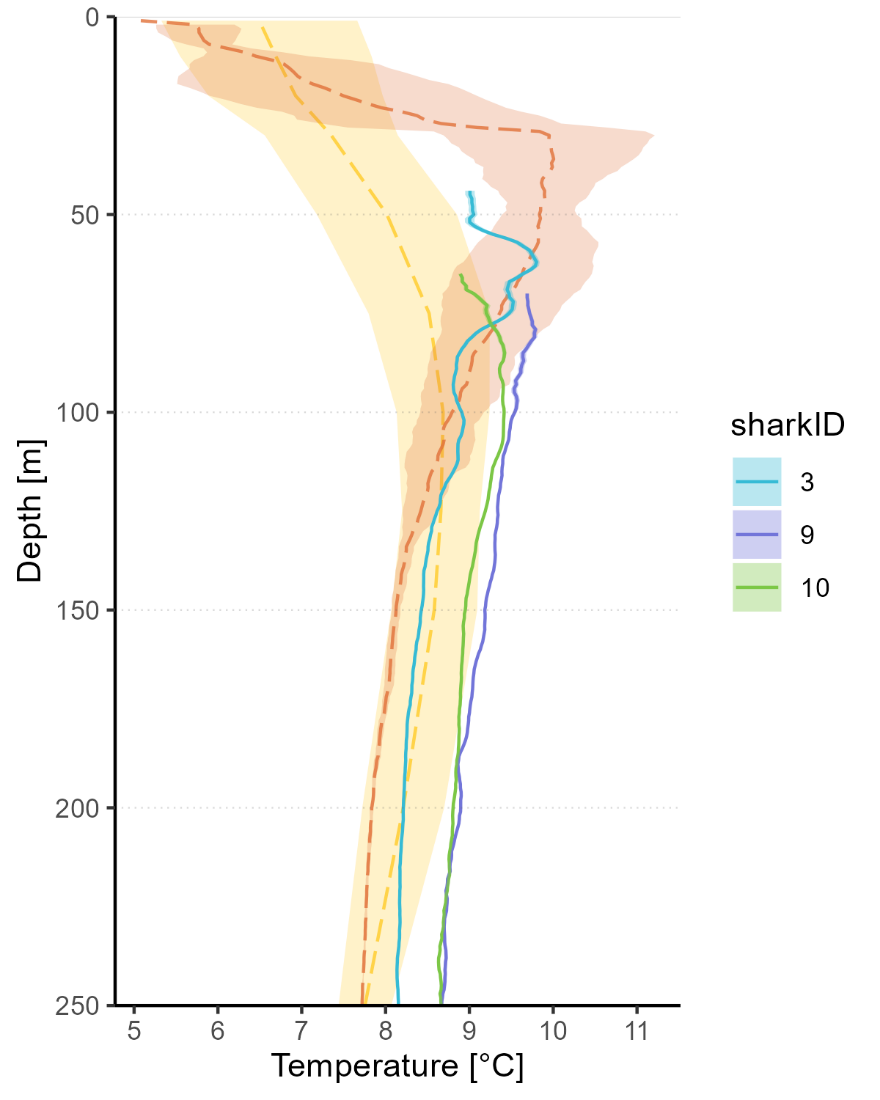


Figure S2: Depth-temperature profiles for the hydrographic stations and from the tracking data of spurdog (*Squalus acanthias* Linnaeus, 1758). Hydrographic stations Utsira (yellow) and H2 (orange) as well as tracking data from used archival PSATs for sharks 3, 9 and 10 for 2020-2023. Means and standard deviations of temperatures are shown for each meter (for Utsira station data was only available at eleven depths). Archival tracking data based on minutely median temperatures for each depth rounded to the next meter. The y-axis is restricted to 250 m corresponding to the depth for which CTD profiles were available.


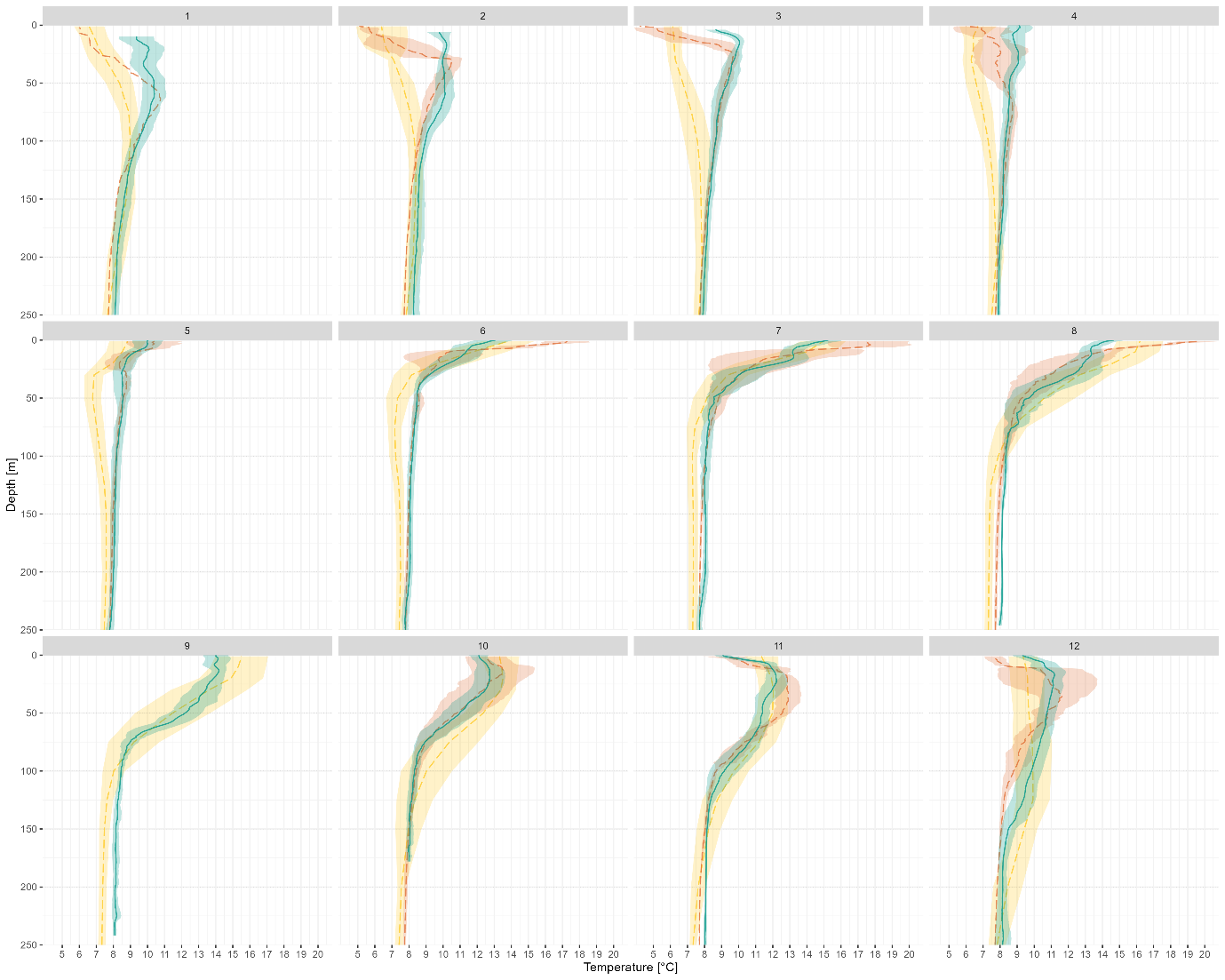


Figure S3: Depth-temperature profiles for the hydrographic stations and from the tracking data of 19 spurdog (*Squalus acanthias*). Hydrographic stations Utsira (yellow) and H2 (orange) as well as tracking data from used archival PSATs (turquoise) for each month (from 1= January to 12= December) within the deployment period (November 2019-October 2023). Means and standard deviations of temperatures are shown for each meter (for Utsira station data was only available at eleven depths). Archival tracking data based on hourly median temperatures for each depth rounded to the next meter. The y-axis is restricted to 250 m corresponding to the depth for which CTD profiles were available.
